# Evidence of Endemic Hendra Virus Infection in Flying-Foxes (*Pteropus conspicillatus*)—Implications for Disease Risk Management

**DOI:** 10.1371/journal.pone.0028816

**Published:** 2011-12-14

**Authors:** Andrew C. Breed, Martin F. Breed, Joanne Meers, Hume E. Field

**Affiliations:** 1 School of Veterinary Science, University of Queensland, Brisbane, Queensland, Australia; 2 Centre for Epidemiology and Risk Analysis, Animal Health and Veterinary Laboratories Agency, Addlestone, Surrey, United Kingdom; 3 Australian Centre for Evolutionary Biology and Biodiversity (ACEBB), and School of Earth and Environmental Sciences, University of Adelaide, North Terrace, South Australia, Australia; 4 Queensland Centre for Emerging Infectious Diseases, Biosecurity Queensland, Department of Employment, Economic Development & Innovation, Coopers Plains, Queensland, Australia; Veterinary Laboratories Agency, United Kingdom

## Abstract

This study investigated the seroepidemiology of Hendra virus in a spectacled flying-fox (*Pteropus conspicillatus*) population in northern Australia, near the location of an equine and associated human Hendra virus infection in late 2004. The pattern of infection in the population was investigated using a serial cross-sectional serological study over a 25-month period, with blood sampled from 521 individuals over six sampling sessions. Antibody titres to the virus were determined by virus neutralisation test. In contrast to the expected episodic infection pattern, we observed that seroprevalence gradually increased over the two years suggesting infection was endemic in the population over the study period. Our results suggested age, pregnancy and lactation were significant risk factors for a detectable neutralizing antibody response. Antibody titres were significantly higher in females than males, with the highest titres occurring in pregnant animals. Temporal variation in antibody titres suggests that herd immunity to the virus may wax and wane on a seasonal basis. These findings support an endemic infection pattern of henipaviruses in bat populations suggesting their infection dynamics may differ significantly from the acute, self limiting episodic pattern observed with related viruses (e.g. measles virus, phocine distemper virus, rinderpest virus) hence requiring a much smaller critical host population size to sustain the virus. These findings help inform predictive modelling of henipavirus infection in bat populations, and indicate that the life cycle of the reservoir species should be taken into account when developing risk management strategies for henipaviruses.

## Introduction

Hendra virus (HeV) and Nipah virus (NiV) are paramyxoviruses of the genus *Henipavirus* with pteropid bats (i.e. flying-foxes; *Pteropus sp.*, Family Pteropodidae) being the primary wildlife reservoir [1]. Evidence of henipavirus infection has been found across the range of pteropid bats from eastern Australia, southeast Asia, Bangladesh, India and Madagascar [2]. Henipavirus infection has also been found to be present in *Eidolon helvum*, a species of fruit bat (Family Pteropodidae) that occurs throughout sub-Saharan Africa [3], [4].

The potential to cross species boundaries from bats to domestic animals and humans causing fatal infection appears to be a consistent feature of henipaviruses wherever they have caused disease (Australia and Asia). Given that over two billion people live in the area where *Pteropus* or *Eidolon* bats are present, even sporadic spillover to humans may result in a significant number of human infections.

Henipaviruses have the potential to infect a wide range of mammalian species, and Hendra virus has spread from flying-foxes to horses in Australia on at least 20 reported separate occasions (five involving horse-human transmission), most recently in 2011 [5], [6], [7]. Seven humans have become infected with HeV via contact with infected horses, resulting in four fatalities [5], [8], [9]. In peninsular Malaysia and Singapore during 1998 and 1999, Nipah virus infected pigs and humans resulting in the death of over 100 humans and the culling of over one million pigs [10]. Since that time, there have also been at least 10 outbreaks of NiV disease in humans in Bangladesh and India, with the resultant death of over 140 people. There is also clear evidence of human-to-human transmission of NiV [11], [12].

In spite of the major health concerns, the knowledge of the epidemiology and ecology of these viruses is limited [1], [13], [14]. How the viruses are maintained in bat populations is not fully understood, nor is how the viruses avoid extinction as their host species become immune. In addition, whether these viruses are predominantly horizontally or vertically transmitted is also uncertain [12], [15], [16], with the apparent viral latency and recrudescence in some human HeV and NiV infections suggesting that henipavirus infection dynamics may differ significantly from the closely related morbilliviruses [17].

A previous study by Plowright *et al.* [14] on the infection dynamics of HeV in the little red flying-fox, *Pteropus scapulatus*, in the Northern Territory of Australia suggested that viral transmission may be predominantly horizontal, with pregnancy and lactation suggested as risk factors for infection. However, Plowright *et al.* [14] sampled multiple colonies over time, leaving the possibility that sampling was not confined to a single population.

Here, we focus on the transmission of HeV in a single colony of flying-foxes over a 25-month period that was approximately 10 km from the location of a spillover of infection to a horse and human in October 2004 [5], [8]. We sampled the spectacled flying-fox, *Pteropus conspicillatus*, a species which is restricted in distribution to the Wet Tropics bioregion of north Queensland [18]. We present data on the infection dynamics of HeV within this flying-fox colony over the 25-month period. We investigated the association of antibody response to the lifecycle stage of the host and the hypothesis that HeV is maintained by episodic infection with periodic virus outbreaks taking place.

## Results

A total of 521 *Pteropus conspicillatus* were sampled over the six sampling sessions with an overall seroprevalence to Hendra virus of 56% (95% C.I. 51–60).

### Seroprevalence

The logistic regression model that included age, sampling session, sex, reproductive status and weight best fitted variance in seroprevalence, thus these variables were analysed in more depth (ΔAIC_c_ = 0.00; ω_i_ = 0.57; all other models ΔAIC_c_>2 and ω_i_<0.2; Appendix 1 in supplementary information). Models that included two- and/or three-way interactions had ΔAICc>5, thus were not investigated further. Weight and forearm length predictor variables were highly correlated (r^2^ = 0.72), but the model that included forearm length and not weight had limited support (Table S1).

#### Temporal variation

**Figure 1:**
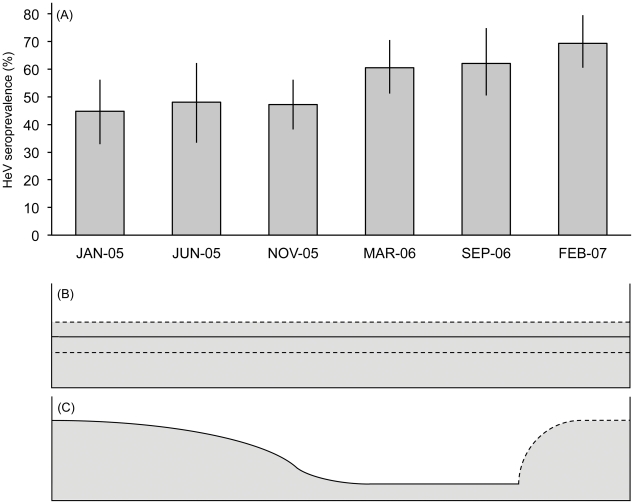
Observed (A) and expected (B, C) patterns of HeV seroprevalence through time. Panel (A) shows observed temporal variation in *Pteropus conspicillatus* seroprevalence (% ±95 CI) over the period of study. Panel (B) schematically represents a theoretical seroprevalence pattern of endemic or persistent infection transmission dynamics (dotted lines represent 95% CIs). Panel (C) schematically represents a theoretical seroprevalence pattern of an acute, self limiting pathogen; a pattern of seroprevalence that could be seen in a population with episodic infection.

Seroprevalence steadily increased over the six sampling sessions from 44.7% in January 2005 to 69.4% in February 2007 (Figure 1; Table S2).

#### Variation with sex and reproductive status

Seroprevalence of female bats did not differ significantly from male bats (female: 58.7%; male: 53.7%; log binomial regression p = 0.25; Table 1). However, pregnant females had a significantly higher seroprevalence than both males and all other female bats (pregnant females: 70.3%; all other bats: 54.6%), and were 1.3 times more likely to have antibodies to HeV than the rest of the bats sampled (95% CI: 1.03–1.61; log binomial regression p<0.05). Bats sampled in early lactation had a significantly higher seroprevalence than male and all other female bats (early lactation: 75.0%; all other bats: 54.9%). Hence, those in early lactation were 1.4 times more likely to have HeV antibodies than the others sampled (95% CI: 1.05–1.78; log binomial regression p<0.05).

**Table 1:** Effect of reproductive status on *Pteropus conspicillatus* HeV seroprevalence.

Sex or reproductive status	*n*_seropositive_/*n*_sampled_	Seroprevalence (%)	Relative risk	Lower 95%CI	Upper 95%CI	P-value
(1) Female	121/206	58.74	1.095	0.939	1.277	0.248
Male	169/315	53.65	1			
(2) Pregnant	26/37	70.27	1.288	1.029	1.613	0.027
All not pregnant	264/484	54.55	1			
(3) All lactating	34/51	66.67	1.224	0.991	1.511	0.060
All not lactating	256/470	54.47	1			
(4) Early lactation	15/20	75.00	1.366	1.048	1.781	0.021
All not early lactation	275/501	54.89	1			

Relative risk of the four group comparisons uses (1) female, (2) pregnant, (3) all lactating and (4) early lactation as the reference group to all sampled bats. P-values for log binomial regression shown.

When seroprevalence was compared among the reproductive categories of adult females, pregnant bats and those in early lactation had a significantly higher seroprevalence than non-reproductive adult females (pregnant: 26/37, 70.3%; non-reproductive adult females: 19/39, 48.7%; Fisher's exact test p<0.05; early lactation: 15/20, 75.0%; Fisher's exact test p<0.05). The seroprevalence of those in late lactation was not significantly higher than non-reproductive females (late lactation: 20/32, 62.5%; non-reproductive adult females: 19/39, 48.7%; Fisher's exact test p = 0.178).

#### Variation with age

**Figure 2:**
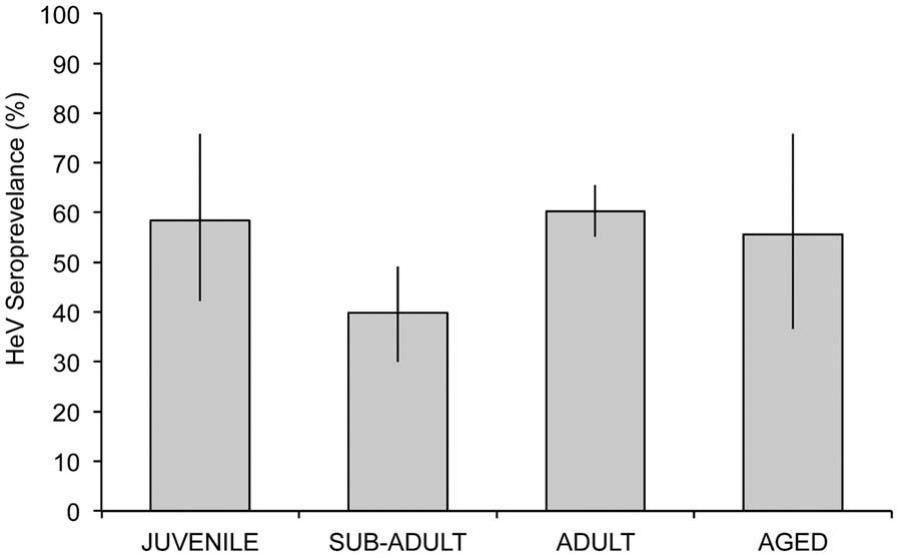
*Pteropus conspicillatus* HeV seroprevalence (% ±95 CI) across age categories. The observed seroprevalence pattern of a higher seroprevalence of juveniles (58.3%) than sub-adults (39.9%), and a correspondingly higher seroprevalence in adults (60.3%) suggests predominantly horizontal transmission of virus and that maternal transfer of HeV antibodies to juveniles likely occurs.

Seroprevalence was highest in bats of the adult category (60.3%), followed by juveniles (58.3%), while sub-adults had significantly lower seroprevalence (39.8%) than both adults (logistic regression p<0.001) and juveniles (logistic regression p<0.05; Figure 2; Table S3).

#### Variation with bodyweight and forearm length

Seroprevalence did not show statistically significant variation with bodyweight and forearm length although a non-significant linear trend was observed between seroprevalence and bodyweight (Table S4).

### Antibody titre levels

Hendra virus antibody titre levels were determined by serial dilution of the sera in the virus neutralisation test. Significant differences in titre levels were found according to sampling session (Kruskal-Wallis test p<0.0001), bodyweight (Kruskal-Wallis test p<0.0001), pregnancy status (Wilcoxon test p<0.001) and sex (Wilcoxon test p<0.01; Table S5). Age, forearm length and lactation status were not significant risk factors for antibody titre level (Kruskal-Wallis and Wilcoxon tests p>0.05).

Antibody levels varied greatly across the sampling sessions with animals sampled in January 2005 having a median titre of 15 (mean rank 25.7) followed by consistent increases to a peak median titre of 80 (mean rank 205.6) in September 2006, followed by a drop to 30 (mean rank 58.8) in February 2007, the final sampling session.

Individuals of greatest bodyweight (>850 g) had the lowest antibody titres with a median of 20 (mean rank of 113.3). Females had significantly higher antibody titres than males (median titres 40 and 20; mean ranks 161.2 and 134.2, respectively) with the highest titres observed for pregnant females (median titre 80; mean rank 197.2). Those in any stage of lactation did not show a significantly higher antibody titre than the rest of the bats sampled.

## Discussion

Previous studies have suggested that henipaviruses are maintained in flying-fox populations through episodic infection in a metapopulation structure, and do not persist endemically within a single population [14], [19] (See Figure 1, panel C). Our findings do not support this hypothesis, but support an alternative pattern of endemic infection in the population. This endemic infection dynamic is consistent with a study on viral excretion of NiV in *Pteropus lylei* in Thailand, where seasonal excretion of virus was observed to occur from the same small colonies each year [12].

Our findings on HeV antibody titre levels show a peak in September 2006 (median titre = 80; mean rank = 205.6) when all adult female bats sampled were at a late stage of pregnancy. This is plausibly consistent with a “boosted” immune response subsequent to the previous sampling session (March 2006: median titre = 40; mean rank = 73.96). The following sampling session showed a decrease in titre level (February 2007: median titre = 30; mean rank = 58.8), but an increase in seroprevalence from 62.1% in September 2006 to 69.4%. This finding is consistent with a period of increased viral transmission during late pregnancy that had resolved by the time the majority of females were lactating, as evidenced by the consequent increased seroprevalence. Indeed Pourrut *et al.*, [20] have suggested that altered immune function in late pregnancy may cause a transient surge in viral replication of filoviruses in African fruit bats.

Our finding that late pregnancy and lactation were risk factors for HeV seropositivity are concordant with results presented by Plowright *et al.* [14] on *P. scapulatus*. Furthermore, the reproductive cycle in other bat species has been linked to seropositivity and viral activity of filoviruses, coronaviruses, lyssaviruses and astroviruses [20], [21], [22]. Our study identified the highest seroprevalence in the first few weeks of lactation, indicated by a seroprevalence of 75.0% in females carrying pups (Figure 2). Our study also supports the conclusions of Field (unpublished data) and Plowright *et al.* [14] that maternal transfer of HeV antibodies to juveniles likely occurs, evidenced by the higher seroprevalence of juveniles (58.3%) than sub-adults (39.9%), and a correspondingly higher seroprevalence of adults (60.3%). These findings are consistent with horizontal transmission of the virus, however the observed seroprevalence pattern does not preclude the occurrence of vertical transmission. Vertical transmission may contribute to viral persistence in bat populations, and there is evidence that vertical transmission of HeV occurs from experimental infection studies of flying-foxes and guinea pigs [15] and from natural infection in wild flying-foxes (Field unpublished data) [16]. Numerous viruses can be transmitted both horizontally and vertically (e.g. transplacentally), including human polyoma virus, bovine viral diarrhoea virus, feline leukaemia virus and parvoviruses (porcine, canine and feline) [17].

For females to be classified as adult in our study they must have shown signs of prior lactation (i.e. enlarged nipples; figure 2), and hence a previous pregnancy and lactation. Our finding that HeV seroprevalence in early lactation was significantly higher than adult females that were not pregnant or lactating (early lactation; 75.5%; not pregnant or lactating: 48.7%; p-value = 0.047) is evidence for a decline in HeV seroprevalence in females in the life stage following lactation. Such a decline suggests that detectable immunity to HeV is not long lived in *P. conspicillatus*, and the pattern seen may reflect seasonal variation in response to repeated exposure. This variation is contrary to the assumption that HeV induces long-lived detectable immunity in *P. conspicillatus* and *P. poliocephalus* (e.g. [14]), and suggests that the transmission dynamics of henipaviruses may be different to those of the closely related morbilliviruses. Indeed, the mechanism of survival of henipaviruses at the population level appears more likely to be one of endemic infection, perhaps similar to that found in bovine viral diarrhoea virus, classical swine fever or some herpes viruses utilising persistent infection, or vertical transmission, as found in arenaviruses or retroviruses [17]. These patterns of infection require much smaller critical host population sizes, in contrast to viruses that demonstrate an acute self-limiting episodic infection pattern determined by: a build-up of susceptibles, introduction of virus, and environmental conditions that promote spread (e.g. measles, Newcastle disease virus or canine distemper virus; [17]; See Figure 1, panel B).

A previous serial cross-sectional study by Plowright *et al.* [14] over a 16-month period on little red flying-foxes, *P. scapulatus*, sought to determine the factors that drive HeV spillover. Their study suggested that age, sex, body size, pregnancy, lactation, season and mating behaviour were possible risk factors for HeV infection, and that horizontal transmission was the major mode of transmission between individuals. They also reported a rapid seroprevalence decline between two successive sampling sessions. However, given their sampling at multiple locations, the expansive geographic distribution and highly nomadic nature of *P. scapulatus* [23], it is plausible that they were not sampling the same population over time. In contrast, the species in our study, *P. conspicillatus*, is not a nomadic species and has a restricted distribution to the Wet Tropics of northeast Queensland [18]. Furthermore, our study was conducted within 10 km of a location where an HeV outbreak occurred in October 2004 [5], and we collected samples from a single colony of *P. conspicillatus* on six separate occasions over a 25-month period. Consequently, we are confident that we followed the infection dynamics of a single population of *P. conspicillatus* over the study period. Nonetheless, interpretation of results from studies in wild animal populations should be made with care. Capture of bats by mist-netting provides a statistically non-random sample of the population, and the practicalities of sampling from a roost site of many thousands of individuals also precludes following individuals over time. To counter these issues, we sought to investigate potential bias and confounding effects where possible. Future studies on henipavirus infection dynamics in wild bats may benefit from: permanent marking of individuals to identify possible repeated capture and sampling of some animals; improved diagnostic capabilities to increase the probability of detection of viral shedding; and improved telemetry methods to enhance the understanding of movement of individuals between roost locations.

Our findings do not support the episodic infection hypothesis for HeV persistence in our study population. Rather we suggest that endemic infection of *P. conspicillatus* occurs, perhaps with periodic pulses of viral transmission associated with late pregnancy and early lactation. The consistent increase in seroprevalence over the duration of our study, together with increasing titres over the first five sessions followed by a drop in titre in the last session, also suggest the presence of inter-annual factors may be affecting viral transmission. An increase in viral transmission associated with pregnancy in flying-foxes is plausibly concordant with the temporal pattern of some HeV incidents in horses in Australia [5], and of NiV outbreaks in humans in Bangladesh and India [12]. This pattern suggests that the risk of henipavirus transmission from flying-foxes to domestic animals and/or humans is higher during the gestation period of flying-foxes. Thus, it is plausible that spillover risk may be uniformly spatially distributed wherever pregnant flying-foxes are present. The observed spatial clustering of henipavirus incidents may be confounded by: surveillance intensity (passive surveillance is the only method used, with heightened awareness of disease likely in areas where previous incidents have occurred); variation in flying-fox population density (there is evidence of increasing movement of flying-foxes in eastern Australia into urban and rural areas [24]); variation in horse density and husbandry practices; and as yet unidentified predisposing ecological or environmental factors (e.g. climate).

A scenario of persistent henipavirus infection with viral latency and recrudescence in flying-foxes has been proposed by Field (unpublished data) and Sohayati *et al.*, [25]. Viral latency and recrudescence has also been shown to occur in a human HeV case [26], and at least 12 human NiV cases [27]. This infection dynamic could lead to the endemic infection pattern seen in our study. However, it is plausible that different social structures of host populations (e.g. panmixia, metapopulation, seasonal aggregation) may favour different mechanisms of maintaining infection at the population level (e.g. predominantly horizontal transmission, predominantly vertical transmission, seasonal explosive outbreaks, repeated viral excretion by persistently infected individuals). Consequently, population structures and mechanisms of maintenance of infection may reflect the biology of the host species and level of ecological disruption, rather than only the biology of the virus. As such, it is likely that different host populations may have varying levels of risk of infection spillover to domestic animals and/or humans.

### Conclusions and perspectives

An improved understanding of the infection dynamics of henipaviruses in bat populations should facilitate the development of effective risk management strategies for disease spillover from bats to domestic animals and/or humans. Here we show that HeV infection in a population of *Pteropus conspicillatus* is likely to be endemic rather than episodic, as previously proposed for HeV in flying-foxes. We also present evidence for seasonal viral activity suggesting that immunity to the virus may wax and wane on a seasonal basis. These findings should inform disease risk management and approaches for modelling henipavirus infection in bat populations. If the ongoing threat that these viruses pose to public health is to be mitigated, further work is required to clarify the principal mode(s) of transmission of henipaviruses in bats, and to comprehensively determine how these viruses persist in their reservoir hosts.

## Materials and Methods

### Study site

All study animals were captured and sampled at the Gordonvale roost site (145°46.74′E, 17°4.86′S). This site is in a 4.5 hectare mixed woodland and forest remnant, surrounded by sugarcane plantations and suburban housing. It has been permanently occupied by *Pteropus conspicillatus* for at least ten years, usually containing 40,000 to 60,000 individuals, constituting approximately 20% of the Australian population of this threatened species [18], [28]. This is the closest known flying-fox colony to the property where the HeV spillover event occurred in October 2004 [5]. The Australian population of *P. conspicillatus* is geographically isolated from other populations of this species in northern Papua New Guinea and the Moluccan islands of Indonesia [29].

### Flying-fox capture and sampling

Flying-foxes were caught in mist nets, anaesthetised with isoflurane (Isoflurane, Laser Animal Health Pty Limited) and oxygen via an anaesthetic machine using the protocol of Johnson *et al.* [30] for sampling. Data collected were sex, bodyweight, forearm length, approximate age and reproductive status according to descriptions detailed in Table S6. Animals were marked with coloured acrylic lacquer on their hind claws to prevent resampling within the same session, and were then released at the site of capture after recovery from anaesthesia.

**Figure 3:**
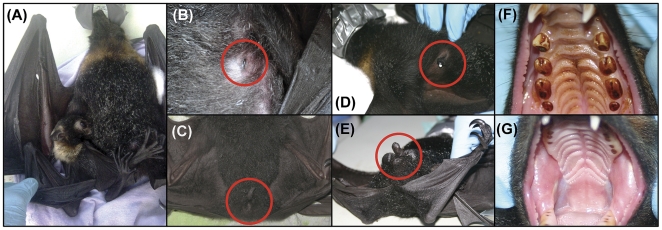
Features for classification of age categories of *Pteropus conspicillatus* used in this study. Age classification features (highlighted by a red circle in B, C, D, E) for both sexes. Key features include: juvenile bats (A) carried by their mother (estimated age 0 to 3 months old); sub-adult bats (B, C) were free flying that lacked signs of sexual maturity, including the lack of enlarged nipples for females (B) or small or non-descended testes for males (C; estimated age 3 months to 2 years); adults bats (D, E, F) showed signs of sexual maturity, including visibly enlarged nipples indicating a previous pregnancy and suckling of young in females (D) or large and descended testes in males (E), and but did not show signs of severe wear on all molar teeth (F; estimated age 2 to 8 years); aged bats (G) showed signs of severe molar wear on all molar teeth, including at least two molars worn to the level of the gingiva (estimated age 8 years and older).

Age classification of sampled bats was performed as described by Hall and Richards ([23]; Figure 3; Table S6). Bats being carried by their mother were classified as juvenile (estimated age 0 to 3 months old; Figure 3, A). Free flying bats that lacked signs of sexual maturity (e.g. small or non-descended testes in males; lack of enlarged nipples in females) were classified as sub-adults (estimated age 3 months to 2 years; Figure 3, B, C). Bats that showed signs of sexual maturity (e.g. large and descended testes in males; visibly enlarged nipples indicating a previous pregnancy and suckling of young in females) but did not show signs of severe wear on all molar teeth were classified as adults (estimated age 2 to 8 years; Figure 3, D, E, F). Bats that showed signs of severe molar wear on all molar teeth, including at least two molars worn to the level of the gingiva, were classified as aged (estimated age 8 years and older; Figure 3, G).

Female bats were further classified according to their reproductive status (Table S6). If a foetus could be palpated while anaesthetised bats were classified as pregnant (this is likely to represent females in the last trimester of pregnancy; [31]). Bats from which milk could be expressed from their teats and were captured carrying a young were classified as early lactating. Bats from which milk could be expressed from their teats, but were not carrying young, were classified as late lactating (*P. conspicillatus* carry their young continuously for the first month after birth, after which time the young are left in a crèche while the females forage for the remainder of lactation [20]). Bats that were not classified into any of the previous three categories, which would include females in early-mid pregnancy, were classified as non-reproductive.

Blood samples were collected by venepuncture of the propatagial vein with a 23 or 25 gauge needle and 1 mL or 3 mL syringe depending on the animal size. Blood was allowed to clot in 2 mL tubes for 24 hours before centrifugation and separation of serum and storage at 4°C.

### Serological tests

Antibody titres to Hendra virus were determined by virus neutralisation test (VNT) according to [32] at the Australian Animal Health Laboratory (Geelong, Victoria); the World Organisation for Animal Health (OIE) reference laboratory for Hendra and Nipah virus diseases. The tests were carried out under biosafety level 4 conditions as Hendra virus is a dangerous human pathogen with a high case fatality rate and for which there is no vaccine or effective treatment [33]. A serum sample was considered positive if it neutralised 100 TCID_50_ of HeV at a dilution of 1∶10 or greater in the VNT, since bat sera at lower dilutions have produced a high rate of toxic or non-specific reactions on neutralisation tests.

### Data analysis

To investigate the association of potential risk factors with serostatus, data were analysed by multivariate logistic regression. We used Akaike's Information Criterion corrected for small sample sizes (AIC*_c_*) for model selection [34]. Models were ranked according to the difference between the best-fitting model and the AICc value of model *i* (ΔAICc). The strength of evidence for alternative models was measured by AICc weights (ω*_i_*). For the potential risk factors identified by multivariate logistic regression to be important in explaining variation in serostatus, we performed log binomial regression analyses to further analyse their associations with serostatus. The relative risk of being seropositive was determined for these predictor variables. Due to the smaller sample sizes, Fisher's exact test was used to investigate the hypothesised effect of reproductive status on serostatus in adult females, where serostatus of non-reproductive adult females was compared with those categorised as either pregnant, early lactation or late lactation.

As titre levels were not assumed to conform to an *a priori* distribution, two measures appropriate for comparing titre data among groups where serial dilution of sera produce logarithmic dilutions were used. These measures were the median titres with an interquartile range, to indicate statistical dispersion, and the mean rank titres, which indicates the mean rank value for the titres of animals within a particular category when all animals are ranked according to titre level. Subsequently, non-parametric models were fitted to the data. For risk factors with two levels, a simple Wilcoxon test was performed. For risk factors with three or more levels, a Kruskal-Wallis test was performed.

### Ethics Statement

All animal work followed the guidelines of the American Society of Mammalogists guidelines [31] and the National Health and Medical Research Council of Australia [32]. The study was approved by the Animal Ethics Committee of the Queensland Department of Primary Industries and Fisheries (Permit number FN 47/2003-1) and the Queensland Parks and Wildlife Service (Permit number WISP03721106).

## Supporting Information

Table S1 Model selection results for the effects of risk factors and the seroprevalence. The model selection statistics are: number of parameters in model (K), Akaike's information criterion corrected for small sample sizes (AIC_c_), difference between model *i* and the model with the smallest AIC_c_ (ΔAIC_c_), AIC_c_ weights (ω_i_) and evidence ratios (ω_i_/ω_j_). Only models with ΔAIC_c_<5 are shown.
(DOC)

Table S2 *Pteropus conspicillatus* HeV seroprevalence for six sampling sessions over a 25-month period. Relative risk is calculated against the first sampling session using log binomial regression analysis.
(DOC)

Table S3 Effect of age on *Pteropus conspicillatus* HeV seroprevalence. Relative risk is calculated against the sub-adult category, using log binomial regression analysis.
(DOC)

Table S4 Univariate log binomial regression analysis for HeV seroprevalence and flying-fox body size (forearm length) and bodyweight. Relative risk compared to categories: forearm length 175 mm or more and bodyweight 850 g or more respectively.
(DOC)

Table S5 Hendra virus antibody titre levels in *P. conspicillatus* according to sampling session, age, forearm length, bodyweight, sex and reproductive status. Only bats returning a positive test result for HeV antibodies are included in the analysis.
(DOCX)

Table S6 Description of variables and categories used in this study.
(DOC)
