# Supplementary material for: Evidence of Endemic Hendra Virus Infection in Flying-Foxes (Pteropus conspicillatus)—Implications for Disease Risk Management
Source: PLoS One. 2011 Dec 14;6(12):e28816. doi: 10.1371/journal.pone.0028816 (PMC3237542; doi:10.1371/journal.pone.0028816)
Supplement: Table S1 — Model selection results for the effects of risk factors and the seroprevalence. The model selection statistics are: number of parameters in model (K), Akaike's information criterion corrected for small sample sizes (AICc), difference between model i and the model with the smallest AICc (ΔAICc), AICc weights (ωi) and evidence ratios (ωi/ωj). Only models with ΔAICc<5 are shown. (DOC) [file pone.0028816.s001.doc]

Table S1. Model selection results for the effects of risk factors and the seroprevalence. The model selection statistics are: number of parameters in model (K), Akaike’s information criterion corrected for small sample sizes (AIC_c_), difference between model *i* and the model with the smallest AIC_c_ (ΔAIC_c_), AIC_c_ weights (ω_i_) and evidence ratios (ω_i_/ω_j_). Only models with ΔAIC_c_ < 5 are shown.

| **Risk factor models** | **K** | **AIC_c_** | **ΔAIC_c_** | **ω_i_** | **ω _i_/ω_j_** |
| --- | --- | --- | --- | --- | --- |
| Seroprevalence ~ sampling session + age + sex & adult female reproduction status + bodyweight | 16 | 687.07 | 0 | 0.57 | 1 |
| Seroprevalence ~ sampling session + age + sex & adult female reproduction status | 15 | 689.43 | 2.37 | 0.18 | 0.31 |
| Seroprevalence ~ sampling session + age + sex & adult female reproduction status + forearm length | 16 | 689.54 | 2.47 | 0.17 | 0.29 |
