# Supplementary material for: Evidence of Endemic Hendra Virus Infection in Flying-Foxes (Pteropus conspicillatus)—Implications for Disease Risk Management
Source: PLoS One. 2011 Dec 14;6(12):e28816. doi: 10.1371/journal.pone.0028816 (PMC3237542; doi:10.1371/journal.pone.0028816)
Supplement: Table S2 — Pteropus conspicillatus HeV seroprevalence for six sampling sessions over a 25-month period. Relative risk is calculated against the first sampling session using log binomial regression analysis. (DOC) [file pone.0028816.s002.doc]

Table S2. *Pteropus conspicillatus* HeV seroprevalence for six sampling sessions over a 25-month period. Relative risk is calculated against the first sampling session using log binomial regression analysis.

| **Sampling session** | ***n*_seropositive_ /*n*_sampled_** | **Seroprevalence (%)** | **Relative risk** | **Lower 95%** | **Upper 95%** | **P-value** |
| --- | --- | --- | --- | --- | --- | --- |
| Jan-05 | 34/76 | 44.74 | 1 |  |  |  |
| Jun-05 | 24/50 | 48.00 | 1.063 | 0.761 | 1.485 | 0.721 |
| Nov-05 | 60/127 | 47.24 | 1.048 | 0.807 | 1.360 | 0.727 |
| Mar-06 | 63/104 | 60.58 | 1.402 | 1.026 | 1.916 | 0.034 |
| Sep-06 | 41/66 | 62.12 | 1.459 | 1.008 | 2.111 | 0.045 |
| Feb-07 | 68/98 | 69.39 | 1.805 | 1.259 | 2.588 | 0.001 |
