# Supplementary material for: Evidence of Endemic Hendra Virus Infection in Flying-Foxes (Pteropus conspicillatus)—Implications for Disease Risk Management
Source: PLoS One. 2011 Dec 14;6(12):e28816. doi: 10.1371/journal.pone.0028816 (PMC3237542; doi:10.1371/journal.pone.0028816)
Supplement: Table S3 — Effect of age on Pteropus conspicillatus HeV seroprevalence. Relative risk is calculated against the sub-adult category, using log binomial regression analysis. (DOC) [file pone.0028816.s003.doc]

Table S3. Effect of age on *Pteropus conspicillatus* HeV seroprevalence. Relative risk is calculated against the sub-adult category, using log binomial regression analysis.

| **Age** | ***n*_seropositive_ /*n*_sampled_** | **Seroprevalence (%)** | **Relative risk** | **Lower 95% CI** | **Upper 95% CI** | **P-value** |
| --- | --- | --- | --- | --- | --- | --- |
| Juvenile | 21/36 | 58.33 | 1.465 | 1.02 | 2.101 | 0.038 |
| Sub-adult | 43/108 | 39.81 | 1 |  |  |  |
| Adult | 211/350 | 60.29 | 1.514 | 1.183 | 1.938 | 0.001 |
| Aged | 15/27 | 55.66 | 1.398 | 0.927 | 2.101 | 0.111 |
