# Supplementary material for: Evidence of Endemic Hendra Virus Infection in Flying-Foxes (Pteropus conspicillatus)—Implications for Disease Risk Management
Source: PLoS One. 2011 Dec 14;6(12):e28816. doi: 10.1371/journal.pone.0028816 (PMC3237542; doi:10.1371/journal.pone.0028816)
Supplement: Table S4 — Univariate log binomial regression analysis for HeV seroprevalence and flying-fox body size (forearm length) and bodyweight. Relative risk compared to categories: forearm length 175 mm or more and bodyweight 850 g or more respectively. (DOC) [file pone.0028816.s004.doc]

Table S4. Univariate log binomial regression analysis for HeV seroprevalence and flying-fox body size (forearm length) and bodyweight. Relative risk compared to categories: forearm length 175mm or more and bodyweight 850g or more respectively.

| **Category** | ***n*_seropositive_/*n*_sampled_ (%)** | **Relative risk** | **Lower 95%** | **Upper 95%** | **P-value** |
| --- | --- | --- | --- | --- | --- |
| *Forearm* |  |  |  |  |  |
| < 155 mm | 51/103 (49.51) | 0.8625 | 0.6692 | 1.1117 | 0.2534 |
| 155-165 mm | 47/87 (54.02) | 0.941 | 0.7308 | 1.2119 | 0.6377 |
| 165-175 mm | 130/223 (58.30) | 1.0155 | 0.8341 | 1.2363 | 0.8784 |
| > 175 mm | 62/108 (57.41) | 1 |  |  | 0.4891 |
| *Weight* |  |  |  |  |  |
| < 550 g | 65/141 (46.10) | 0.746 | 0.5952 | 0.935 | 0.011 |
| 550-700 g | 76/130 (58.46) | 0.8851 | 0.7107 | 1.1023 | 0.2758 |
| 700-850 g | 70/122 (57.38) | 0.9216 | 0.7449 | 1.1403 | 0.4524 |
| > 850 g | 79/128 (61.72) | 1 |  |  | 0.0534 |
