# Supplementary material for: Evidence of Endemic Hendra Virus Infection in Flying-Foxes (Pteropus conspicillatus)—Implications for Disease Risk Management
Source: PLoS One. 2011 Dec 14;6(12):e28816. doi: 10.1371/journal.pone.0028816 (PMC3237542; doi:10.1371/journal.pone.0028816)
Supplement: Table S5 — Hendra virus antibody titre levels in P. conspicillatus according to sampling session, age, forearm length, bodyweight, sex and reproductive status. Only bats returning a positive test result for HeV antibodies are included in the analysis. (DOCX) [file pone.0028816.s005.docx]

Table S5. Hendra virus antibody titre levels in *P. conspicillatus* according to sampling session, age, forearm length, bodyweight, sex and reproductive status. Only bats returning a positive test result for HeV antibodies are included in the analysis.

| **Category** | **Sample size** | **Median titre (IQR)** | **Mean rank titre** | **P-value** |
| --- | --- | --- | --- | --- |
| *Date* |  |  |  | < 0.001^a^ |
| Jan-05 | 34 | 15 (30) | 25.73 |  |
| Jun-05 | 24 | 20 (30) | 30.41 |  |
| Nov-05 | 60 | 40 (60) | 61.00 |  |
| Mar-06 | 63 | 40 (60) | 73.96 |  |
| Sep-06 | 41 | 80 (120) | 205.6 |  |
| Feb-07 | 68 | 30 (70) | 58.82 |  |
| *Age group* |  |  |  | 0.852^a^ |
| AD | 211 | 40 (70) | 145.13 |  |
| AG | 15 | 40 (60) | 154.16 |  |
| JU | 21 | 40 (60) | 156.16 |  |
| SA | 43 | 20 (70) | 139.04 |  |
| *Sex* |  |  |  | 0.006^b^ |
| Female | 121 | 40 (60) | 161.23 |  |
| Male | 169 | 20 (30) | 134.23 |  |
| *Pregnant* |  |  |  | 0.001^b^ |
| Pregnant | 26 | 80 (120) | 197.23 |  |
| Not pregnant | 264 | 40 (70) | 140.4 |  |
| *Lactating* |  |  |  | 0.391^b^ |
| Lactating | 34 | 40 (60) | 156.85 |  |
| Not lactating | 256 | 40 (70) | 143.99 |  |
| *Early lactation* |  |  |  | 0.988^b^ |
| Early lactating | 15 | 40 (20) | 145.16 |  |
| Not early lactating | 275 | 40 (70) | 145.51 |  |
| *Weight* |  |  |  | < 0.001^a^ |
| 550 or less | 65 | 40 (60) | 146.46 |  |
| 550-700 | 76 | 40 (140) | 178.98 |  |
| 700-850 | 70 | 40 (70) | 144.56 |  |
| 850 or more | 79 | 20 (30) | 113.32 |  |
| *Forearm length* |  |  |  | 0.491^a^ |
| 155 or less | 51 | 40 (70) | 144.21 |  |
| 155-165 | 47 | 40 (70) | 145.85 |  |
| 165-175 | 130 | 40 (60) | 152.09 |  |
| 175 or more | 62 | 20 (30) | 132.46 |  |

IQR = interquartile range; ^a^Kruskal-Wallis test; ^b^Wilcoxon test
