# Supplementary material for: Evidence of Endemic Hendra Virus Infection in Flying-Foxes (Pteropus conspicillatus)—Implications for Disease Risk Management
Source: PLoS One. 2011 Dec 14;6(12):e28816. doi: 10.1371/journal.pone.0028816 (PMC3237542; doi:10.1371/journal.pone.0028816)
Supplement: Table S6 — Description of variables and categories used in this study. (DOC) [file pone.0028816.s006.doc]

Table S6. Description of variables and categories used in this study.

| **Predictor variables** | **Description** | ***n*** |
| --- | --- | --- |
| *Sex* |  |  |
| Male | Identified as male on external examination | 206 |
| Female | Identified as female on external examination | 315 |
| *Age* |  |  |
| Juvenile | Young that were carried by their mother; estimated age 0 to 3 months old | 36 |
| Sub-adult | Animals that were caught free flying and lacked signs of sexual maturity i.e. small or non-descended testes for males and lack of enlarged nipples for females; estimated age 3 months to 2 years | 108 |
| Adult | Animals that showed signs of sexual maturity i.e. large and descended tests in males and visibly enlarged nipples indicating a previous pregnancy and suckling of young in females, but without signs of severe wear on all molar teeth; estimated age 2 to 8 years | 350 |
| Aged | Animals with signs of severe molar wear on all molar teeth including at least two molars worn to the level of the gingiva; estimated age 8 years and older | 27 |
| *Adult female reproductive status* |  |  |
| Non-reproductive | Females classified as adult (as per above) but not identified as pregnant or lactating | 39 |
| Pregnant | Animals in which a foetus could be detected by abdominal palpation while anaesthetised. This is likely to represent females in the last trimester of pregnancy (Towers and Martin, 1985) | 36 |
| Early lactating | Animals from which milk could be expressed from their teats and were captured carrying a young | 20 |
| Late lactating | Animals from which milk could be expressed from their teats but were not carrying a young | 32 |
| *Sampling session* |  |  |
| Jan-05 | Flying-foxes sampled at study site over 8 days | 76 |
| Jun-05 | Flying-foxes sampled at study site over 8 days | 50 |
| Nov-05 | Flying-foxes sampled at study site over 8 days | 127 |
| Mar-06 | Flying-foxes sampled at study site over 8 days | 104 |
| Sep-06 | Flying-foxes sampled at study site over 8 days | 66 |
| Feb-07 | Flying-foxes sampled at study site over 8 days | 98 |
| *Bodyweight* | Measured to the nearest gram using digital electronic scales | 521 |
| *Forearm length* | Measured in millimetres from the proximal tip of the olecranon process to distal tip of the carpus | 521 |
